# Quantum Dots Reveal Shifts in Organic Nitrogen Uptake by Fungi Exposed to Long-Term Nitrogen Enrichment

**DOI:** 10.1371/journal.pone.0138158

**Published:** 2015-09-14

**Authors:** Nicole A. Hynson, Steven D. Allison, Kathleen K. Treseder

**Affiliations:** 1 Department of Botany, University of Hawai‘i Manoa, 3190 Maile Way, Honolulu, HI, 96822, United States of America; 2 Department of Ecology and Evolutionary Biology, University of California Irvine, Irvine, CA, 92697, United States of America; Chengdu Institute of Biology, CHINA

## Abstract

Anthropogenic nitrogen (N) enrichment can alter N dynamics associated with decomposing plant litter. However, it is unclear to what extent these alterations occur via microbial effects (e.g., changes in gene regulation, physiology, or community composition) versus plant litter effects (e.g., changes in composition of N and C compounds). To isolate microbial effects from plant litter effects, we collected plant litter from long-term N fertilized and control plots, reciprocally inoculated it with microbes from the two treatments, and incubated it in a common field setting for three months. We used quantum dots (QDs) to track fungal uptake of glycine and chitosan. Glycine is a relatively simple organic N compound; chitosan is more complex. We found that microbial and litter origins each contributed to a shift in fungal uptake capacities under N fertilization. Specifically, N fungi preferred glycine over chitosan, but control fungi did not. In comparison, litter effects were more subtle, and manifested as a three-way interaction between litter origin, microbial origin, and type of organic N (glycine versus chitosan). In particular, control fungi tended to target chitosan only when incubated with control litter, while N fungi targeted glycine regardless of litter type. Overall, microbial effects may mediate how N dynamics respond to anthropogenic N enrichment in ecosystems.

## Introduction

Numerous laboratory and field studies have reported that environmental conditions can alter the composition and function of microbial communities [1–4]. For instance, changes in nitrogen availability, soil moisture, and temperature can each elicit shifts in microbial community structure in parallel with shifts in N dynamics, decomposition rates, or soil CO_2_ respiration [1–4]. Moreover, a growing number of laboratory studies have demonstrated a link between community composition and function: direct manipulation of microbial community composition often alters N and C dynamics [5–10]. This link could occur because microbes vary in their physiological capacity to take up and transform various N compounds [11–16]. Thus, it is possible that shifts in the microbial community can be partly responsible for alterations in ecosystem functions under environmental change, if these shifts alter the relative abundance of taxa with different physiological capabilities [17]. In addition, physiological shifts can occur within taxa via acclimation or adaptation [18, 19].

A “common garden” approach can be employed to isolate these microbial effects from the immediate influence of the environment. Indeed, several laboratory studies have found that microbial communities isolated from different environments can function differently from one another even when grown in a common environment [17, 20–24]. Nevertheless, when examining microbial contributions to ecosystem dynamics, field-based experiments are particularly worthwhile, because they can integrate complex environmental conditions that are often difficult to replicate in a laboratory. Only a minority of studies have compared the functions of different microbial communities by transplanting them into a common *field* setting [17, 25–28]. For the most part, they have done so by encasing microbes within “cages” of nylon mesh that has pores large enough to allow the passage of solutes and gases, but small enough to restrict the movement of fungi and bacteria [27]. These field studies have documented that ecosystem-level dynamics like N mineralization, nitrification, and decomposition vary among communities transplanted to a common setting [17, 25–28]. However, the changes in microbial physiology that drive these differences remain largely unexamined, primarily because they are often difficult to assess in the field.

In this study, we focus on fungal uptake of specific organic N compounds, because N uptake is a well-defined physiological process with a number of known consequences for ecosystem function [29]. For example, uptake of organic N can lead to immobilization of N in microbial biomass, which limits its availability for other organisms [30, 31]. Alternatively, microbes can mineralize their acquired organic N compounds and secrete excess N as ammonium, thereby augmenting N availability [32, 33]. Nitrogen availability is important, because net primary productivity of plants is N-limited in many ecosystems [34, 35].

We focused on N enrichment as an element of the environment that can alter microbial function. Anthropogenic N deposition is common in Southern California, with many natural ecosystems receiving more than 25 kg N ha^-1^ y^-1^ [36]. Nitrogen enrichment can affect microbes via numerous mechanisms, including alterations in nutrient contents of the plant litter they decompose [37]. For example, in a grassland in Southern California, N additions elicit increases in litter N [17]. In a common garden experiment in this ecosystem, microbes from the N-fertilized plots were associated with different decomposition rates [17] and enzymatic efficiencies [22] compared to those from the control plots. Specifically, after six months of decomposition, N microbes were associated with faster decomposition when they were incubated in the N-fertilized plots versus the control plots [17]. In addition, extracellular cellulases and hemicellulases tended to be more efficient when microbes were placed on litter from their home treatment [22]. Here, we examined organic N uptake in this same experiment.

Fungi are sensitive to N enrichment; their community composition frequently changes upon chronic exposure to excess N [38–42]. In fact, N additions in our study site significantly altered fungal community composition on standing plant litter [43]. Specifically, taxa within the Davidiellaceae, Tremellaceae, and Didymosphaeriaceae displayed marked declines in relative abundance in the N fertilized plots compared to the control plots, while species in Pleosporaceae were significantly more abundant under N fertilization (J. Martiny, unpubl. data). Since fungi are often responsible for the transformation of relatively complex organic material [44], shifts in their communities might have important consequences for ecosystem function. In this study, we addressed the question: How does N enrichment influence uptake capacities for organic N in fungi, and to what extent does this occur via changes in litter versus changes in fungal origin?

Accordingly, we tested two hypotheses related to organic N uptake by fungi. Specifically, we investigated the capacity of fungi to internalize organic N from the environment, measured as the rate of uptake per unit fungal biovolume over 24 hours. First, we hypothesized that N fungi would prefer glycine over chitosan to a greater extent than would control fungi (Hypothesis 1). We expect this response because complex organic N compounds like chitosan are more costly to metabolize than are simpler organic compounds like glycine [45]. Fungi should be more likely to invest in uptake of complex organic N compounds where N is less available (e.g., control conditions). Second, we hypothesized that both fungal communities would regulate uptake capacities of glycine versus chitosan depending on litter origin (Hypothesis 2). Specifically, we predicted that control fungi should display higher uptake capacities for chitosan on litter from control plots than on litter from the N-fertilized plots. Conversely, N fungi should have higher uptake capacities for glycine on litter from N-fertilized plots than on litter from the control plots.

Quantum dots (QDs) are a relatively new technique that can be used to quantify uptake of specific organic N compounds in microbial communities in field samples [46]. Quantum dots are nanoscale semiconductors that fluoresce in pure, bright colors when exposed to ultraviolet light [47, 48]. Typically, they are 5 to 50 nm in diameter and are constructed of a heavy metal or copper core surrounded by a zinc sulfide shell [49, 50]. We used specialized QDs that were coated with a protective polymer that was embedded with carboxyl groups. We conjugated relatively simple (glycine) or complex (chitosan) organic N compounds to the carboxyl groups. Fungi can take up QD-labeled organic N through membrane transporters including ADP1-encoded transporters [51]. Quantum dot-labeled glycine and chitosan are largely taken up intact [46]. We then tracked uptake of each by using confocal microscopy to count the number of QD-labeled compounds within individual fungal cells, as described in Whiteside et al. [46].

## Methods

### Field site

We tested our hypotheses in an N-fertilization experiment established in a coastal grassland in Orange County, California USA (33° 44’ N, 117° 42’ W, 365 m elevation). The N-fertilization experiment is explained in detail by Allison et al. [17]. Briefly, the grassland is dominated by exotic annual grasses and forbs as well as the native perennial grass *Nassella pulchra* [52]. It receives a mean annual precipitation of 325 mm y^-1^, mostly as rainfall between October and April. Mean annual temperature is 17°C. Permission was granted by the Irvine Ranch Corporation for access and use of this field site.

The N-fertilization experiment commenced in February 2007. Plots were established in eight blocks. In each block, one plot was N-fertilized, and the other remained unmanipulated as a control. Each plot was 3.3 x 9.3 m. Each year, N fertilized plots received soluble calcium nitrate (20 kg N ha^-1^) prior to the growing season plus 100-day release calcium nitrate (40 kg N ha^-1^) during the growing season.

First, we collected plant litter to use as a decomposition substrate. We collected litter from four haphazardly located 0.07 m^2^ quadrats in each control plot and N plot on 29 June, 2 July, and 14 September 2010. (Multiple collection dates were required, because deep-rooted annual forbs senesced later in the year than did the other plant groups.) Litter from all plots within each treatment was pooled and homogenized by hand.

Second, we constructed “microbial cages” in which we decomposed the plant litter [sensu 27]. Microbial cages are litterbags that are constructed of nylon membrane with 0.45 μm pores. Water, nutrients, and unusually small bacteria can pass through these pores, but fungi and most bacteria cannot. We placed 2 g air-dried litter in each cage, and sterilized the assembled cages with at least 22 kGy gamma irradiation. Sterility was verified by placing subsamples of irradiated litter in 50 mL sterile tubes with either lysogeny broth (for bacterial growth, Fisher Scientific, Pittsburgh PA) or potato dextrose broth (for fungi growth; Becton, Dickinson and Company; Franklin Lakes, NJ), shaking at 37°C (12 hours for bacteria and three days for fungi). We then plated out 100 μl of the media on lysogeny broth (or potato dextrose media) plates. We performed the same protocol with a positive control (i.e., non-irradiated litter) to confirm that this procedure facilitated colony growth.

Third, we collected microbial inoculum from control and N-fertilized plots to add to the microbial cages. On 30 November 2010, three haphazardly located litter samples (~5 g each) from each of the eight N and eight control plots were collected by hand. Litter samples were combined within each treatment to generate two batches of microbial inoculum: “control microbes” and “N microbes”. The inoculum litter was air dried, ground in a Wiley mill (1 mm mesh), and added in 50 mg aliquots to bags containing sterilized litter.

### Experimental design

Fungi can acquire N from two major sources in the environment: plant litter and soil. Nitrogen fertilization in this field site increases N availability in both sources [17]. Prior to decomposition, litter from the N-fertilized plots had significantly higher concentrations of total N (i.e., %N), cellulose, and hemicellulose compared to litter from control plots (S1 Table)[17]. In contrast, C:N ratios, lignin concentrations, and sugar concentrations were significantly lower in the N litter than the control litter (S1 Table)[17]. Concentrations of total C (i.e, %C), protein, starch, and fat did not differ significantly by litter origin (S1 Table)[17]. Litter chemistry was originally analyzed by Allison et al. [17] from the same litter used in the current study. They determined total C, total N, and C:N by elemental analysis, and the other fractions by near infrared spectroscopy.

For the current study, we isolated the effects of these changes in litter chemistry by decomposing control (“control litter”) and N-fertilized plant litter (“N litter”) in the control plots only. In other words, soil N availability was the same for each treatment. Accordingly, we placed four microbial cages (one of each treatment) in each of eight control plots. The cages were placed within standing grass litter. One corner of each microbial cage was tethered to the soil surface, and the remainder rested on the standing grass litter. This way, the orientation of the microbial cage matched that of the standing litter. Each cage contained either control or N litter inoculated with control or N microbes in a factorial design. Thus, there were 32 microbial cages total. Microbial cages were incubated in the control plots for three months, from 15 December 2010 to 3 March 2011. Upon collection, the litter from each microbial cage was divided into subsamples. Each of three subsamples received QDs conjugated to a different organic N substrate: glycine or chitosan, or unconjucated QDs (details below). The N compounds represent relatively simple and complex organic N, respectively.

### Preparation of quantum dots

**Fig 1:**
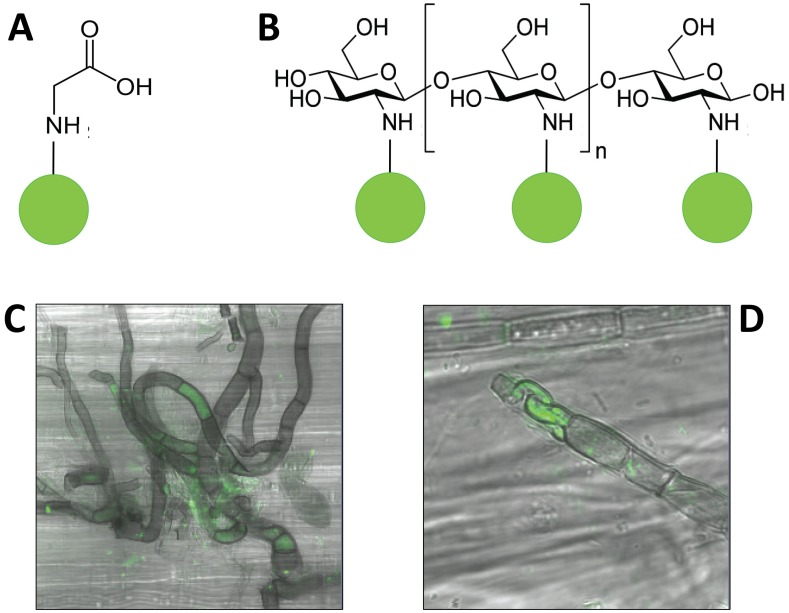
Configurations of QD-glycine (A) and QD-chitosan (B); and QD-chitosan in the interior of fungal hyphae from the N-fertilized community growing on N-fertilized litter (C), and control microbes on control litter (D). A, B: Each organic N compound was conjugated to a green QD via a strong covalent bond between the amino group in the organic N compound and the carboxyl group embedded in the shell of the QD. C, D: We examined each sample in a confocal microscope, and performed three-dimensional scans to verify that the QDs (green) were located in the interior of the hyphae.

QD-labeled compounds were prepared according to a modified Whiteside et al. [53] method. Specifically, QDs were conjugated to the amino group of glycine or the amino groups of chitosan (Fig 1). We used commercial 3 nm diameter green (530 nm emission) carboxyl-terminated QDs (Carboxyl-Functionalized eFluor® 525NC, catalog # 91-6364-33, eBioscience, San Diego, CA USA). Stock QD solutions were conjugated with individual compounds in a 33:1 ratio (substrate:QD) using the binding activator 1-ethyl-3-(3-dimethylaminopropyl) carbodiimide hydrochloride (EDC). After 2 h of conjugation, each solution underwent dialysis against 2 L of sterile water and was then centrifuged through Millipore Amicon™ Ultra Centrifugal Filters to isolate the conjugated QD compounds. Sterile water was added to the conjugated QD compounds to attain 0.1 mmol QD compounds. We also prepared QD-references by subjecting them to the same procedure, but without adding the binding activator. Thus, the QD-references were embedded with carboxyl groups only; they were not conjugated with organic N.

### Laboratory incubations

Three 2 mg subsamples of litter from each microbial cage were added to separate wells of a 96 well plate. We added to each subsample 100 ml of sterile water plus 100 ml of QD-reference, QD-glycine, or QD-chitosan solution. The plate was sealed and incubated in the dark at 20°C for 24 h, after which the plate was stored at –80°C.

### Quantum dot uptake

We used a Zeiss LSM 710 (Quasar spectral detection unit) confocal microscope to measure uptake of QD compounds. First, after calibrating for autofluorescence, we manually scanned random points of each subsample reading only the green wave length (530 nm) corresponding to our green QDs. Where QDs were present in fungal hyphae, we performed a three-dimensional scan to confirm that the QDs were located in the interior of the hyphae. We then used raster image correlation spectroscopy (RICS) to quantify the concentration of QD compounds in the surrounding 1 μm^3^ biovolume of hyphae exactly as detailed in Whiteside et al. [46]. In this manner, we conducted 3–5 measurements within each subsample. The resulting QD concentrations indicated the uptake capacity, per unit biovolume, of fungi that target each compound. Because these measurements were time-intensive, we did not analyze every subsample. Instead, we assessed uptake of QD-glycine and QD-chitosan in five microbial cages of each treatment, and QD-references in six randomly chosen microbial cages.

Uptake rates of QD-references provide a baseline indicating the affinity of fungi for either QDs themselves, or for the carboxyl groups embedded in their shells [53]. For this study, the uptake rate of QD-references averaged 433 ±114 QDs μm^-3^ d^-1^ (mean ±1SE). If QD-glycine or QD-chitosan were taken up more than the QD-references, then the fungi were likely targeting the organic N compound. Therefore, we calculated *relative* QD uptake for each sample by taking the quotient of the QD uptake rate in that sample (i.e., number of QD-glycine or QD-chitosan μm^-3^ d^-1^) divided by the average QD uptake rate for the QD-references (i.e., 433 QD-references μm^-3^ d^-1^). Relative QD uptake values that were greater than 1 indicated uptake of QD-conjugated N.

### Statistics

To test our hypotheses, we used a factorial analysis of variance with a split-plot design. The dependent variable was relative QD uptake. The between-plot variables were microbe origin (control versus N microbes), litter origin (control versus N litter), and their interaction (microbe*litter). The within-plot variables were compound type (QD-glycine or QD-chitosan) and its factorial interaction with microbe origin and litter origin (compound*microbe, compound*litter, and compound*microbe*litter). Where appropriate, Tukey post hoc tests were used for pairwise comparisons between groups.

Hypothesis 1 would be supported by a significant interaction between compound type and microbe origin, so that relative uptake of QD-glycine was significantly higher than QD-chitosan for N fungi, but not for control fungi. A significant interaction between compound type, microbial origin, and litter origin would support Hypothesis 2, if relative uptake of QD-chitosan by control fungi were higher when growing on control litter than on N litter, and relative uptake of QD-glycine by N fungi were higher when growing on N litter than on control litter.

Furthermore, to determine if uptake of QD-glycine or QD-chitosan was significantly greater than uptake of QD-references in each treatment, we conducted a series of two sample t-tests. Each test compared uptake capacities (as QDs μm^-3^ d^-1^) of the QD-references with the uptake capacities in a given compound*microbe*litter treatment.

For all tests, data were ranked to avoid errors associated with non-normality and heterogeneity of variances. Systat 13 was used to perform the tests [54]. Differences were considered significant if P < 0.05 and marginally significant if P < 0.10.

## Results

### Quantum dot uptake

**Fig 2:**
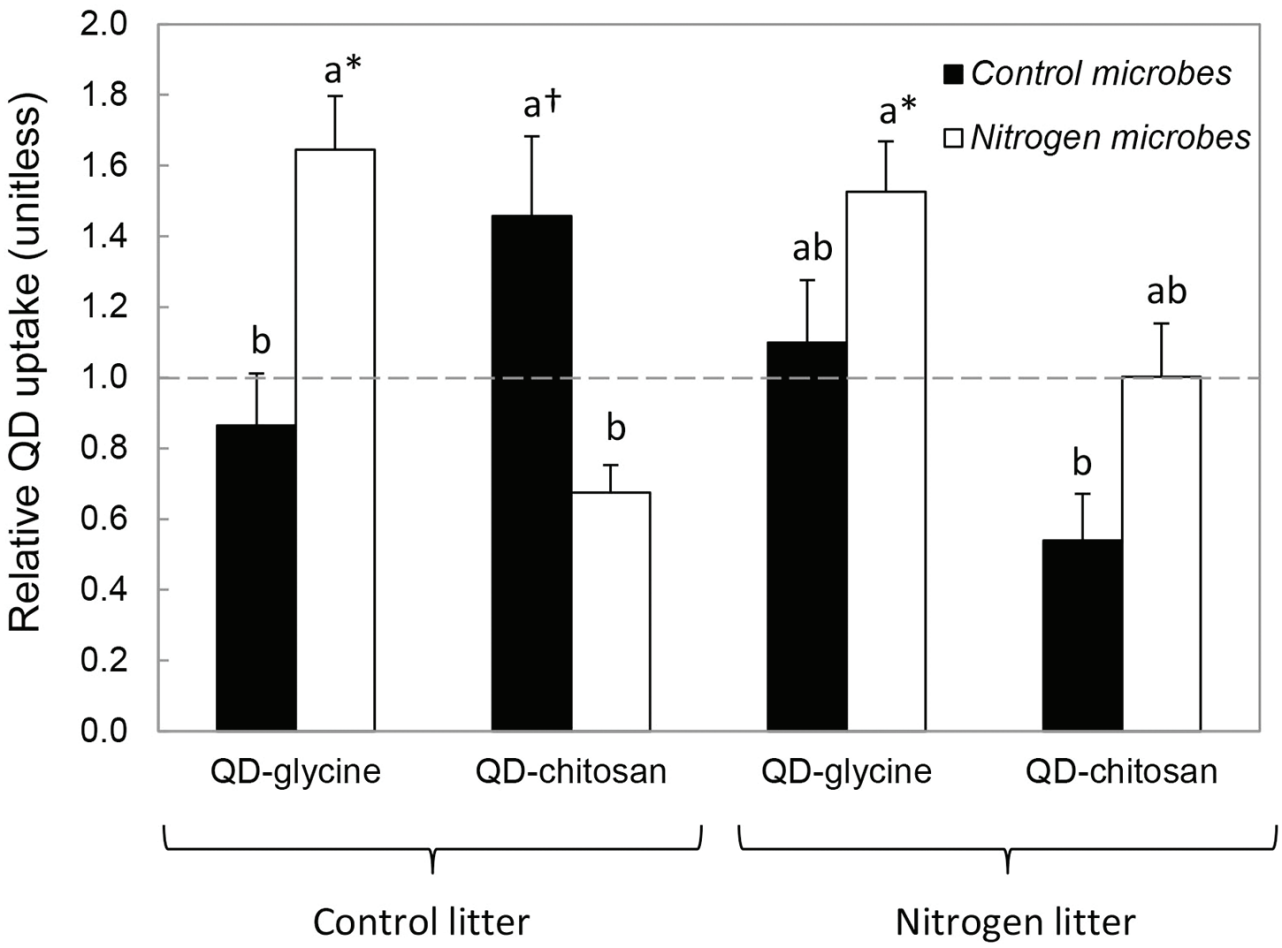
Relative uptake capacity of QD-glycine and QD-chitosan. Relative uptake capacity is QD uptake capacity of QD-compounds (as QDs μm^3^ d^-1^) divided by QD uptake capacity of QD-references. Microbes from control versus N-fertilized plots were added to control versus N-fertilized litter in microbial cages, in a reciprocal transplant design. All microbial cages were decomposed in control plots. Microbe origin, litter origin, and compound type interacted significantly to influence relative QD uptake (P = 0.003). Uptake values are relative to uptake rates of QD-references. Relative QD uptake > 1 (dashed line) indicates that fungi were targeting the organic N compound, rather than the QD or the carboxyl groups embedded in the QD surface. Symbols indicate significant (*) or marginally significant (†) uptake of QD-glycine or QD-chitosan relative to QD-references. Treatments with different letters are significantly different from one another (P < 0.05). Bars are means ±1SE of 5 replicates.

Fungal hyphae internalized QDs in every sample (e.g., Fig 1). Furthermore, fungi appeared to be targeting the QD-conjugated organic N under certain circumstances. Specifically, uptake rates of QD-glycine or QD-chitosan were significantly (or marginally significantly) greater than QD-references in three treatments: QD-glycine in N microbes on control litter (Fig 2, t = 2.50, P = 0.017), QD-glycine in N microbes on N litter (t = 2.16, P = 0.029), and QD-chitosan in control microbes on control litter (t = 1.60, P = 0.072). When compared across litter and microbial treatments, relative uptake of QD-glycine was significantly greater than that of QD-chitosan (Table 1, F_1,1_ = 11.840, P = 0.003).

**Table 1:** Relative uptake capacity of QD compounds by group.

Group	Relative QD uptake^a^
Litter origin (P = 0.322)	
Control litter	1.16 ±0.12 (20)
N litter	1.04 ±0.11 (20)
Microbe origin (P = 0.006)	
Control microbes	0.99 ±0.11 (20) a
N microbes	1.21 ±0.11 (20) b
Organic N compound (P = 0.003)	
QD-chitosan	0.92 ±0.11 (20) a
QD-glycine	1.28 ±0.10 (20) b
Microbe origin * litter origin (P = 0.013)	
Control microbes on control litter	1.16 ±0.16 (10) ab
Control microbes on N litter	0.82 ±0.14 (10) a
N microbes on control litter	1.16 ±0.18 (10) ab
N microbes on N litter	1.26 ±0.13 (10) b
Organic N compound * litter origin (P = 0.148)	
QD-chitosan by control litter	1.07 ±0.17 (10)
QD-chitosan by N litter	0.77 ±0.12 (10)
QD-glycine by control litter	1.26 ±0.16 (10)
QD-glycine by N litter	1.31 ±0.13 (10)
Organic N compound * microbe origin (P = 0.004)	
QD-chitosan by control microbes	1.00 ±0.20 (10) a
QD-chitosan by N microbes	0.84 ±0.10 (10) a
QD-glycine by control microbes	0.98 ±0.12 (10) a
QD-glycine by N microbes	1.59 ±0.10 (10) b

^a^QD uptake capacity (as QDs μm^-3^ d^-1^) compared to QD-references, as means ±1SE (n). Groups with different letters are significantly different (P < 0.05).

Hypothesis 1 was supported. Control and N fungi differed significantly in their preferences for glycine versus chitosan (Table 1, compound*microbe interaction, F_1,1_ = 11.592, P = 0.004). In particular, across litter treatments, N fungi preferred QD-glycine over QD-chitosan, while control fungi acquired each at similar rates (Table 1).

These preferences also depended on litter quality on which the fungi were inoculated. We observed a significant interaction between compound, microbe origin, and litter origin (Fig 2, F_1,16_ = 12.344, P = 0.003). When grown on control litter, control fungi took up more QD-chitosan than QD-glycine (Fig 2). In contrast, the same fungal community—control fungi—did not noticeably target either compound when incubated with N-fertilized litter. Nitrogen fungi, though, were not as responsive to litter type. They acquired QD-glycine regardless of litter type, and they took it up at similar rates in control and N litter (Fig 2). Likewise, their relative uptake rates of QD-chitosan were minimal on both litter types. These results partially support Hypothesis 2, with the exception of our prediction that N fungi would target less QD-glycine on control litter than on N litter.

Across all treatments, N fungi displayed significantly higher relative QD uptake than did control fungi (Table 1, F_1,1_ = 10.083, P = 0.006). There was no significant difference in relative QD uptake between control and N litter across treatments (F_1,1_ = 1.046, P = 0.322). In addition, we observed no significant interaction between compound type and litter type (F_1,1_ = 2.314, P = 0.148).

## Discussion

### Microbial origin

We found that N enrichment altered fungal uptake capacities for organic N, and that changes in the microbial community and alterations in plant litter chemistry each contributed to this shift in fungal physiology. Fungi in the coastal grassland were exposed to N fertilization for 3.75 years before they were transplanted to control litter and N litter. Across litter types, uptake of QD-glycine was higher in N fungi than in control fungi (Table 1). These differences were evident even after the fungi were incubated in a common field setting (control plots) for three months. This pattern suggests that the changes in the fungi elicited by N fertilization [43] influenced their physiological functions. Individuals with a relatively high uptake capacity for simple N may have efficiently acquired added N that had been incorporated into the plant litter. In contrast, individuals with membrane transporters optimal for transport of complex organic N may have been at a disadvantage. Complex organic N can be more expensive to use than simpler organic N, because fungi need to invest more in the production and maintenance of enzymes to depolymerize the complex N, in addition to membrane transporters to take up the products [55, 56]. This trade-off could have selected for glycine users, but not chitosan users, in response to N fertilization.

Shifts in fungal community composition may have contributed to the significant effect of microbe origin on organic N uptake. In the same microbial cages that we used for measurements of organic N uptake, fungal community composition differed significantly between microbial origins (P = 0.001, J. Martiny, unpubl. data). The fungal taxa that contributed most to the differences between microbial origins were from Davidiellaceae (12.9% of composition differences between control and N microbes), Pleosporaceae (10.6%), Tremellaceae (4.0%), and Didymosphaeriaceae (3.9%). Pleosporaceae are saprotrophic and pathogenic sac fungi, and this family was significantly more abundant in the N microbes compared to the control microbes. The other three families declined significantly. (Davidiellaceae contains endophytes, pathogens, and saprotrophs; Tremellaceae includes parasites and pathogens; and Didymosphaeriaceae are primarily saprotrophs). In contrast, fungal community composition did not change significantly when inoculated to control and N litter and field incubated over our experimental period (P = 0.117, J. Martiny, unpubl. data). Differences in fungal community composition between the control and N microbes might have contributed to the observed differences in organic N uptake. For example, Pleosporaceae taxa, which were more prominent in the N microbes than the control microbes, might have possessed greater uptake capacity for glycine. Changes in gene frequency within populations of fungal taxa are another possible mechanism.

### Litter origin

Plant litter also appeared to affect organic N uptake. In this study, we isolated litter effects by incubating each fungal community on control versus N litter. Neither fungal community acquired significant amounts of QD-chitosan on N litter (Fig 2). In contrast, on control litter, the control microbes took up QD-chitosan, albeit marginally significantly. Because N-fertilized litter from this experiment has higher N concentrations than does control litter [17], it may not have been necessary for control microbes to invest extra resources in complex organic N uptake under these conditions [56].

Aside from this interaction between microbe origin and litter origin on relative uptake of QD-chitosan (Table 1, Fig 2), litter effects were subtle at most. For example, litter origin by itself did not significantly alter relative QD uptake, nor did it significantly mediate fungal preferences for QD-glycine versus QD-chitosan. Yet, microbe origin significantly altered these variables. Within this common garden experiment, changes in fungal community structure may have been more important than alterations in litter chemistry. We note, however, that we did not test for effects of changes in soil N availability as all microbial cages were decomposed in control plots.

### Organic N compounds

Chitosan is present in the necromass of fungi and arthropods [57, 58], so it is an alternate source of N that fungi may exploit to meet their demands for N. However, it is a relatively large, complex molecule [58] that must be hydrolyzed (internally or externally) before its N can be incorporated into new compounds [57, 59, 60]. In contrast, glycine is a precursor in protein and purine biosynthesis, with no prior transformation required [61, 62]. In the current study, relative uptake of QD-chitosan was generally lower than that for QD-glycine (Table 1), which indicates that fungi invested less in its uptake overall. Nevertheless, investment in chitosan uptake capacity may be advantageous where N is less available, such as in litter from the control plots (S1 Table). Indeed, Alster et al. [22] noted that potential activity of extracellular chitin-degrading enzymes was higher in cages with control microbes compared to those with N microbes, which is consistent with our findings of significant chitosan uptake in control microbes.

Uptake of organic N by fungi is challenging to measure under field conditions, and has rarely been quantified under N fertilization. Mycorrhizal uptake of chitosan tends to decline with N fertilization in a boreal forest [46], which is broadly consistent with the current study. Moreover, extracellular chitinase activity, which indicates investment by microbes in chitin or chitosan acquisition, often decreases in soils with N enrichment [38, 56, 63, 64]. Economic principles suggest that fungi should reduce investment in complex organic N acquisition where simple N is more available [56], and previous field studies support this notion. The current study extends our knowledge by demonstrating that one of the associated physiological changes—uptake capacity—results from an inherent trait of the microbial community and not simply a function of litter chemistry.

### Caveats

This study has certain caveats that must be considered when interpreting the results. First, the compounds that were conjugated to the QDs could have been modified by extracellular enzymes or other microbes before uptake by fungi. Therefore, the uptake capacities that we report for each compound are potentially influenced by the activity of the community’s extracellular enzymes. In addition, Whiteside et al. [46] noted that transformations of QD-conjugated substrates are minimal (< 4%) within 24 hours, which was the length of the incubation period. Second, our study is relatively short term (3 months), so we do not know how long the physiological shifts in the microbial community will remain. Yet, Alster et al. [22] found that differences between N microbes and control microbes in their extracellular enzyme activities were maintained for at least the first year of the transplant.

### Conclusion

We used QDs and microbial cages to distinguish microbial versus litter effects of N enrichment on fungal uptake capacities for organic N. We found that N enrichment altered the inherent physiological function of the fungi, leading to an increase in uptake capacities for glycine, a relatively simple organic N compound, in favor of chitosan, which is more complex. In this way, N availability affected organic N uptake via changes in microbial gene regulation, physiological traits, or community composition. In addition, the chemical composition of plant litter affected organic N uptake—the control fungi invested more in complex N uptake when they were growing on litter with lower N concentrations (*i*.*e*., control litter) than higher N concentrations (*i*.*e*., N litter). Our findings suggest that fungal effects can mediate rates of N uptake in ecosystems, in addition to changes in plant litter owed to an increase in N availability. In other words, the fungi from the two treatments were not functionally redundant. Shifts in microbial community composition under global change are a common occurrence [1–4], and our results suggest that physiological processes like organic N uptake can change in concert, with potential consequences for ecosystem function.

## Supporting Information

S1 Table Initial chemical content of litter before decomposition. (PDF)
