# Supplementary material for: Quantum Dots Reveal Shifts in Organic Nitrogen Uptake by Fungi Exposed to Long-Term Nitrogen Enrichment
Source: PLoS One. 2015 Sep 14;10(9):e0138158. doi: 10.1371/journal.pone.0138158 (PMC4569051; doi:10.1371/journal.pone.0138158)
Supplement: S1 Table — (PDF) [file pone.0138158.s001.pdf]

**Table S1. Initial chemical content of litter before decomposition[a]**

| Chemical fraction | Nitrogen litter | Control litter | n | P-value |
|---|---|---|---|---|
| C:N (g/g) | 33.06 ±0.40 | 41.68 ±0.83 | 10 | <0.001 |
| %C | 35.92 ±0.18 | 35.95 ±0.13 | 10 | 0.884 |
| %N | 1.09 ±0.02 | 0.87 ±0.02 | 10 | <0.001 |
| Protein (% dry mass) | 6.14 ±0.14 | 6.05 ±0.13 | 8 | 0.658 |
| Cellulose (% dry mass) | 40.92 ±0.36 | 39.66 ±0.29 | 8 | 0.017 |
| Hemicellulose (% dry mass) | 24.73 ±0.33 | 22.30 ±0.35 | 8 | <0.001 |
| Lignin (% dry mass) | 7.04 ±0.1 | 7.71 ±0.07 | 8 | <0.001 |
| Sugars (% dry mass) | 4.06 ±0.19 | 4.55 ±0.11 | 8 | 0.043 |
| Starch (% dry mass) | 3.14 ±0.17 | 3.30 ±0.18 | 8 | 0.518 |
| Fat (% dry mass) | 1.77 ±0.05 | 1.92 ±0.05 | 8 | 0.054 |

[a]Data correspond to "initial litter" column in Table 2 of Allison et al. [17]
